# A mouse model of hereditary coproporphyria identified in an ENU mutagenesis screen

**DOI:** 10.1242/dmm.029116

**Published:** 2017-08-01

**Authors:** Ashlee J. Conway, Fiona C. Brown, Robert O. Fullinfaw, Benjamin T. Kile, Stephen M. Jane, David J. Curtis

**Affiliations:** 1 Australian Centre for Blood Diseases, Monash University and Clinical Haematology, Alfred Health, Melbourne 3004, Australia; 2 Porphyria Reference Laboratory, Biochemistry Department, Royal Melbourne Hospital, Parkville 3050, Australia; 3 ACRF Chemical Biology Division, The Walter and Eliza Hall Institute of Medical Research, Parkville 3052, Australia; 4 Central Clinical School, Monash University, Melbourne 3004, Australia

**Keywords:** Ethyl-N-nitrosourea, Hereditary coproporphyria, CPOX, Anaemia

## Abstract

A genome-wide ethyl-N-nitrosourea (ENU) mutagenesis screen in mice was performed to identify novel regulators of erythropoiesis. Here, we describe a mouse line, RBC16, which harbours a dominantly inherited mutation in the *Cpox* gene, responsible for production of the haem biosynthesis enzyme, coproporphyrinogen III oxidase (CPOX). A premature stop codon in place of a tryptophan at amino acid 373 results in reduced mRNA expression and diminished protein levels, yielding a microcytic red blood cell phenotype in heterozygous mice. Urinary and faecal porphyrins in female RBC16 heterozygotes were significantly elevated compared with that of wild-type littermates, particularly coproporphyrinogen III, whereas males were biochemically normal. Attempts to induce acute porphyric crises were made using fasting and phenobarbital treatment on females. While fasting had no biochemical effect on RBC16 mice, phenobarbital caused significant elevation of faecal coproporphyrinogen III in heterozygous mice. This is the first known investigation of a mutagenesis mouse model with genetic and biochemical parallels to hereditary coproporphyria.

## Abstract (precis)

**Summary:** A mouse mutagenesis model of hereditary coproporphyria has significant genetic and biochemical parallels to that of the human condition.

## INTRODUCTION

The porphyrias are a collection of hereditary disorders characterised by a deficiency in one or more of the enzymes within the haem biosynthesis pathway (Kauppinen, 2005). Hereditary coproporphyria (HCP) is an acute hepatic disorder that stems from a dominantly inherited mutation in the gene encoding the sixth haem enzyme coproporphyrinogen III oxidase (*CPOX*) (Siegesmund et al., 2010). This enzyme is responsible for the conversion of coproporphyrinogen III into protoporphyrinogen IX in the mitochondria of haem-synthesising cells. More than 65 mutations within the *CPOX* gene have been characterised in humans so far, encompassing missense, nonsense, frameshift and insertion/deletion mutations, each with variable penetrance, enzyme functionality and clinical manifestation (Bissell and Wang, 2015). Clinically, porphyria usually emerges in adolescence with acute attacks triggered by factors that activate hepatic enzymes, such as fasting, alcohol, sulphonamide antibiotics, and hormones such as progesterone (Bissell and Wang, 2015). Biochemically, HCP presents with a marked increase in porphyrin precursors, such as porphobilinogen (PBG), as well as porphyrins, notably coproporphyrinogen III, which accumulate and are detected in urine and faeces in high concentrations during episodic attacks, but can be normal or only marginally elevated during latent periods. Avoidance of known triggers is so far the only approach to managing the acute hepatic porphyrias. Symptoms can be alleviated with substances that inhibit haem biosynthesis, such as glucose loading (Anderson et al., 2005) or the administration of haem arginate (Besur et al., 2014). A liver transplant has been shown to be curative in some subtypes of porphyria (Singal et al., 2014).

Animal models representing the hereditary porphyrias have been generated for all porphyria subtypes except for HCP and aminolevulinic acid dehydratase deficiency porphyria (ADP) (Richard et al., 2008; Homedan et al., 2015; Medlock et al., 2002; Phillips et al., 2011). These animal models have been vital for the understanding of mitochondrial biosynthesis pathways and the development of novel gene replacement therapies. Through an ethyl-N-nitrosourea (ENU) mutagenesis screen to identify novel regulators of erythropoiesis, we have generated a novel mouse strain harbouring a nonsense mutation in *Cpox* (W373X), resulting in a microcytic hypochromic red blood cell phenotype. This mouse strain is the first reported model of hereditary coproporphyria with parallels to the human condition.

## RESULTS

### Characterisation of a mouse strain with microcytic anaemia

**Fig. 1.**
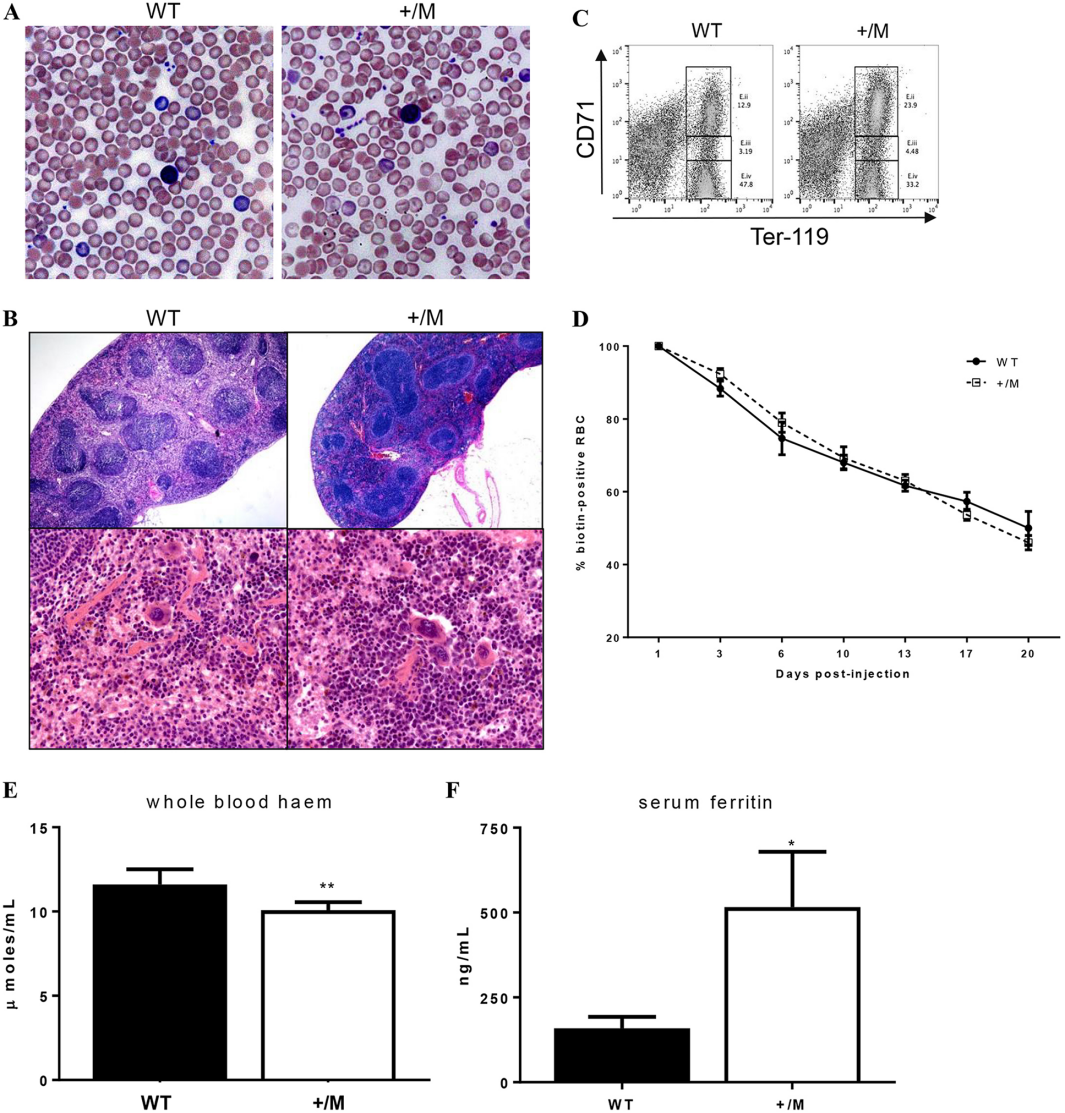
**Phenotype of the RBC16 mouse mutant.** (A) Peripheral blood smears of 7-week-old wild-type (WT) and RBC16 heterozygous (+/M) mice show microcytic hypochromic red cells and prominent target cells. (B) H&E staining of sectioned spleens harvested from 4-month-old wild-type and heterozygous mice at both low (×40) and high (×200) magnification. (C) FACS plot of spleen cells stained with Ter-119 and CD71. Gated Ter-119^+^ CD71^hi^ cells reveal a significant increase in early erythroblast (E.i) populations in the mutant. (D) Red cell half-life measured *in vivo* using biotinylation; *n*=4. Whole blood haem (E) and serum ferritin (F) quantification. Values are mean±s.d; *n*=4. *P<0.05, **P<0.01.

Using ENU mutagenesis to identify novel genes regulating erythropoiesis, we identified a mouse line (RBC16) with a microcytic anaemia (Table 1). Fifty per cent of progeny born from the founder mouse mated with a wild-type mouse displayed a reduced mean corpuscular volume (MCV), indicating that the phenotype was autosomal dominant and fully penetrant. Red cell distribution width (RDW) was markedly increased in heterozygotes, while platelet counts and white cell counts were normal. Peripheral blood smears revealed microcytic hypochromic red cells and abundant target cells (Fig. 1A). RBC16 heterozygotes (+/M) had enlarged spleens with increased red pulp (Fig. 1B) and flow analysis of spleen cells revealed a substantial increase in early erythroblast expansion (Fig. 1C). No notable pathology was identified in the liver (data not shown). Increased spleen size suggested the possibility of haemolysis; however, reticulocyte counts (Table 1) and red cell half-life (Fig. 1D) were both normal. In addition, whole blood haem in the red cells of heterozygotes was found to be significantly reduced (Fig. 1E), while serum ferritin was markedly elevated (Fig. 1F). RBC16 heterozygous mice therefore presented with microcytic anaemia more suggestive of a defect in haem iron than globin chain synthesis.

**Table 1.**
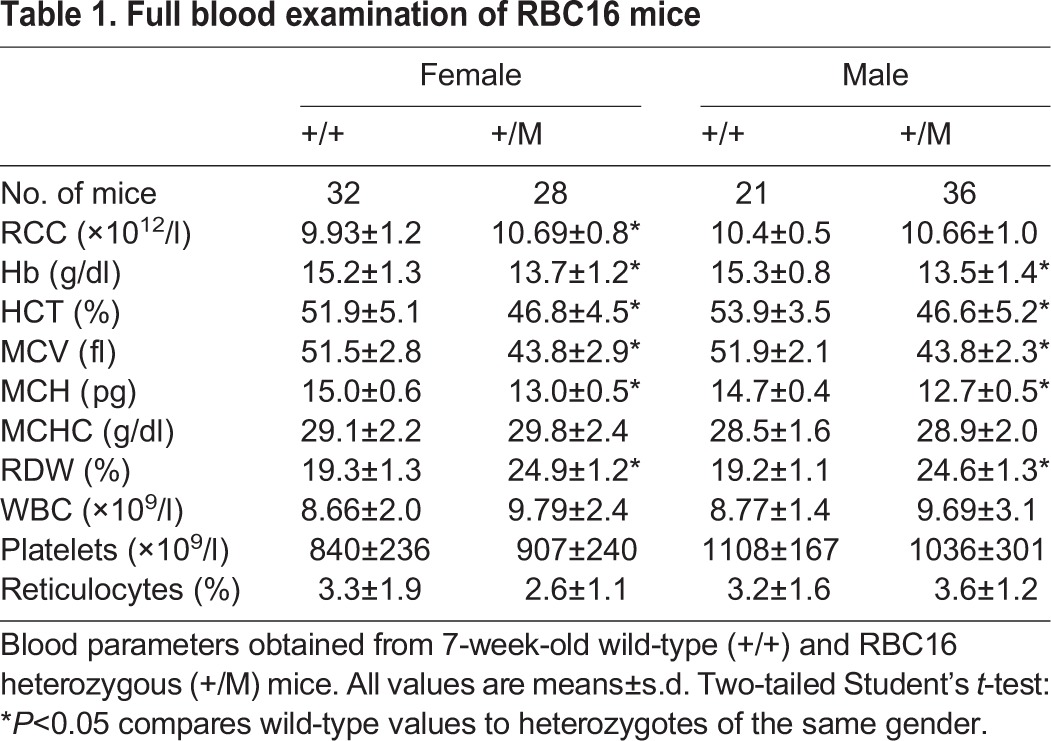
**Full blood examination of RBC16 mice**

### RBC16 mice have a mutation of the haem biosynthetic pathway gene *Cpox*

**Fig. 2.**
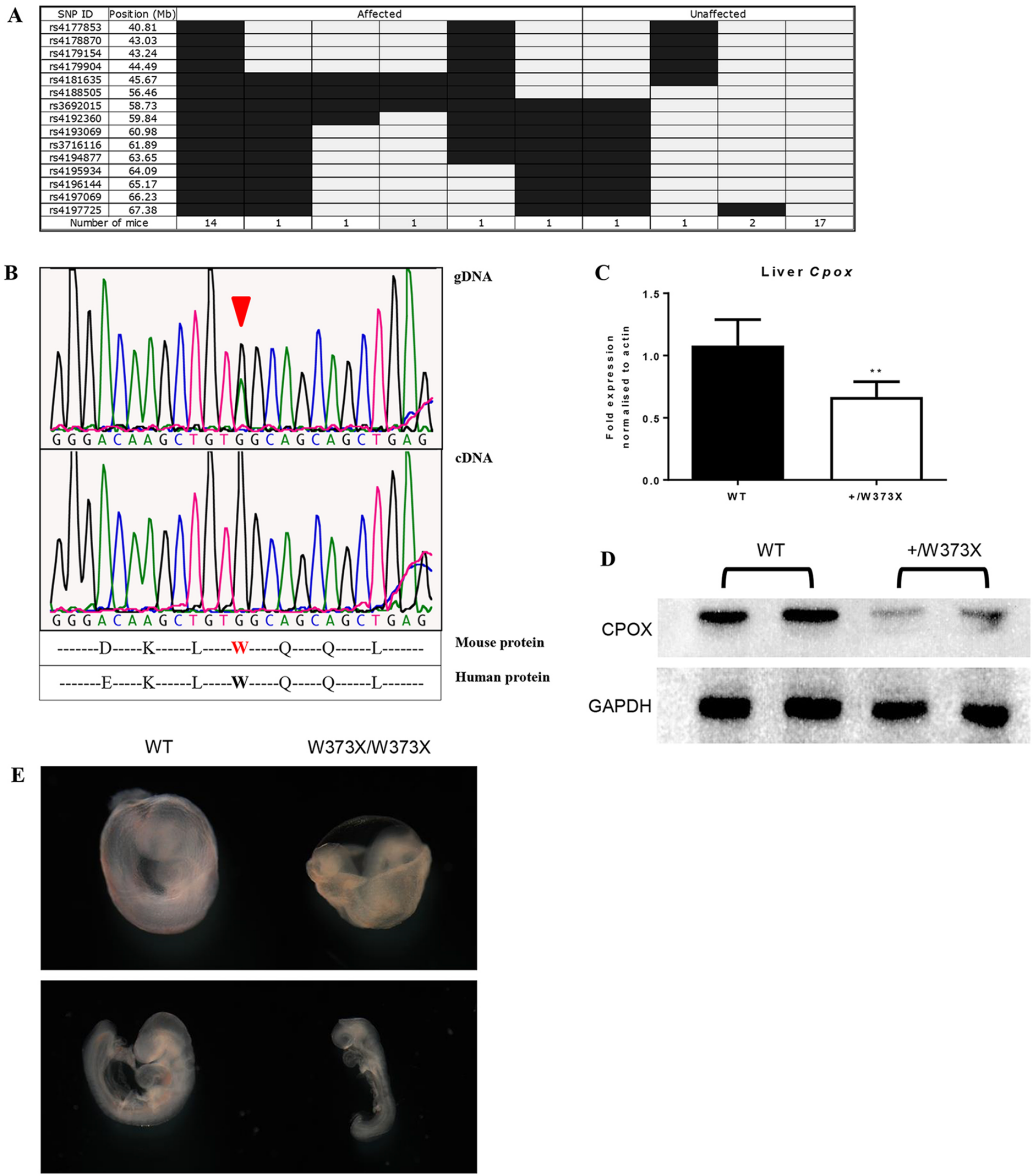
**Characterisation of the W373X mutation.** (A) Fine SNP mapping of 40 mice at 15 SNP markers localised the RBC16 mutation between 56.46 Mb and 60.98 Mb on chromosome 16. Light grey indicates homozygous for Balb/c; dark grey indicates heterozygous for Balb/c and C57BL/6. (B) Sanger sequencing of heterozygous RBC16 gDNA shows the G→A substitution in *Cpox* (arrowhead), whereas in heterozygous cDNA, only the wild-type sequence is visible. (C) Quantitative RT-PCR for *Cpox* mRNA expression in the liver. Values are mean±s.d., *n*=4; **P<0.005. (D) Western blot of the CPOX protein from liver lysates of WT and +/W373X mice. (E) Wild-type (WT) and homozygous (W373X/W373X) embryo littermates dissected at day 9.5, showing yolk sac (top) and whole embryo formation (bottom).

The ENU-induced mutation was mapped by crossing RBC16^+/M^ mice (C57BL/6 background) with wild-type Balb/c mice as previously described (Brown et al., 2013). Genome-wide and fine-mapping of single nucleotide polymorphisms (SNPs) was performed on animals displaying low MCV, which localised the mutation to a region lying between 56.46 Mb and 60.98 Mb on chromosome 16 (Fig. 2A). Custom genome capture of the 3.43 Mb genomic interval was combined with massively parallel sequencing of DNA from a RBC16^+/M^ mouse (based on low MCV) using the Illumina HiSeq platform. This identified a G to A substitution at position c.1118 in exon 5 of *Cpox* (NM_007757.2), predicted to produce an immediate stop codon in place of a highly-conserved tryptophan at amino acid 373 (W373X) (Fig. 2B). Sanger sequencing of genomic DNA extracted from multiple presumed heterozygotes, identified by low MCV, confirmed this mutation. Sanger sequencing of cDNA, generated from the bone marrow of *Cpox*^c.1180G>A^ heterozygotes (denoted as *Cpox^+/W373X^* hereafter), did not detect the nucleotide substitution (Fig. 2B), suggesting that the truncated transcript had undergone degradation. In addition, quantitative real-time (Q)PCR demonstrated a 50% reduction in *Cpox* mRNA expression (Fig. 2C), and western blotting of liver lysates confirmed a similar loss of total CPOX protein (Fig. 2D). Overall, the absence of detectable mRNA and 50% reduction in protein suggested the W373X mutation was a functional null allele.

Intercrossing of heterozygous *Cpox^+/W373X^* mice did not yield a third phenotype in adult progeny, suggesting embryonic lethality of homozygotes. Genotyping of embryos showed that homozygous mutants did not survive beyond embryonic day (E) 9.5 (Table 2). Microscopic examination of E9.5 homozygous embryos showed severe developmental delays, including underdeveloped phalangeal arch structures, lack of prominent forelimb bud, and an open rostral neural tube (Fig. 2E). A vascularised yolk sac was visible in the homozygotes with evidence of blood cell formation, suggesting that homozygous mutants did not die as a result of an inability to produce blood.

**Table 2.**
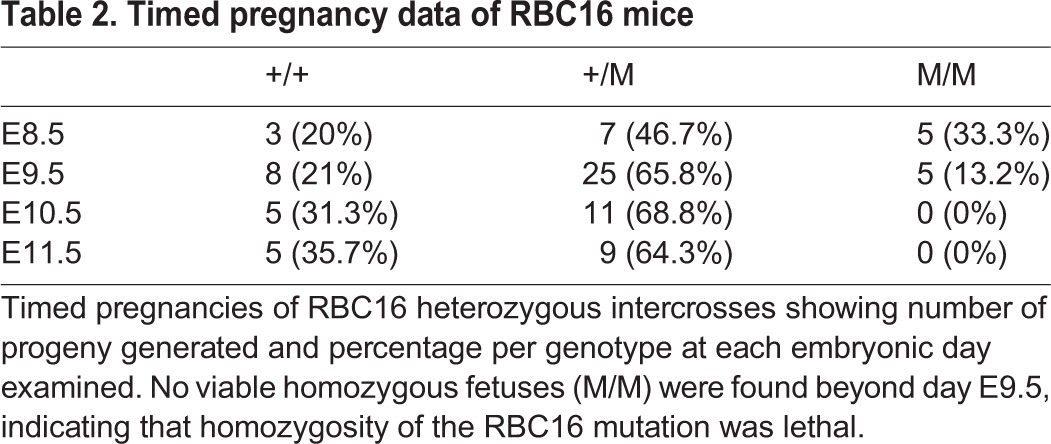
**Timed pregnancy data of RBC16 mice**

### *Cpox^+/W373X^* mice display a mild hereditary coproporphyria phenotype

**Fig. 3.**
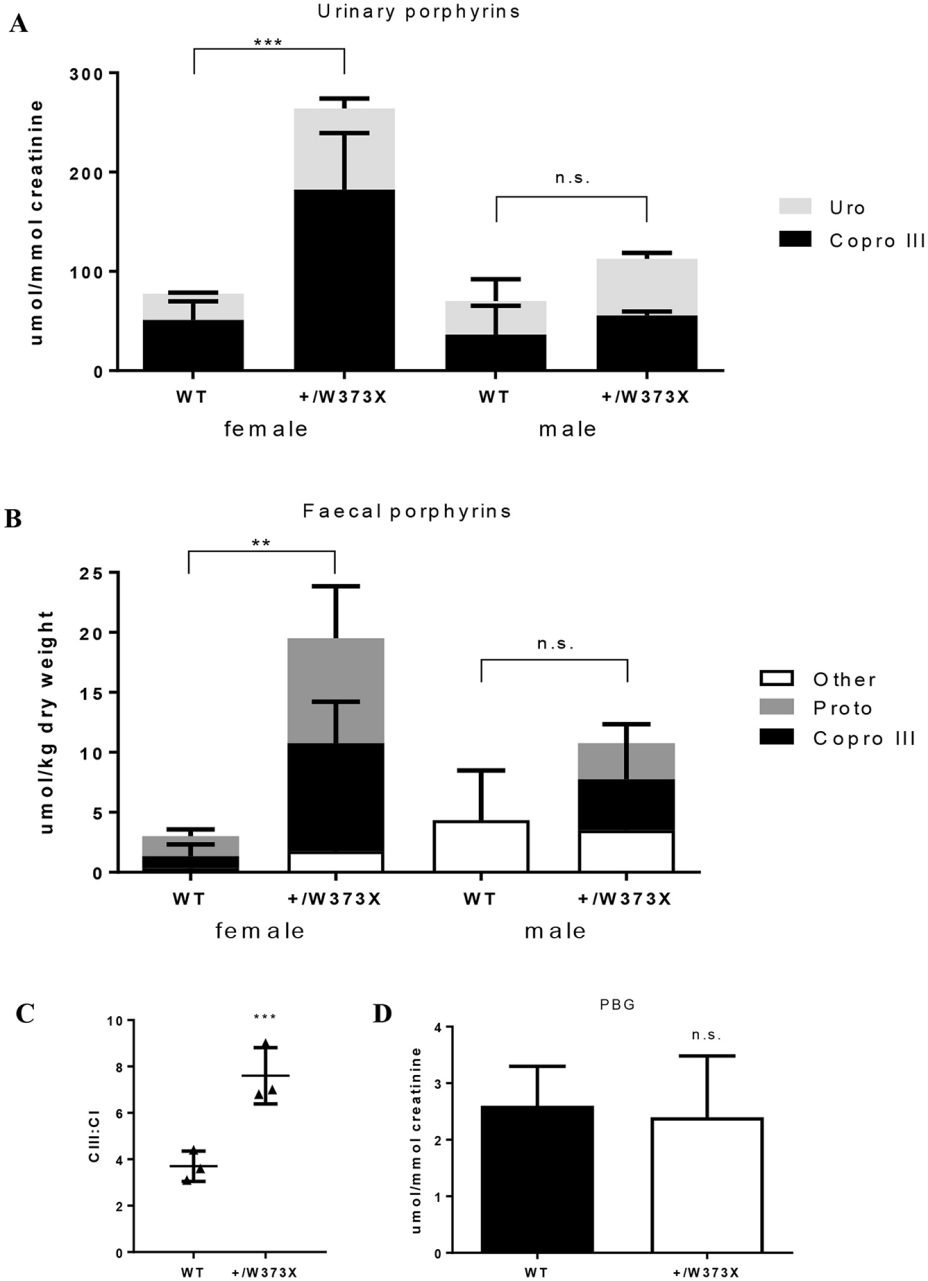
**Porphyrin studies in *Cpox^+/W373X^* mice.** (A) Baseline urinary and (B) faecal porphyrins of 5-month-old RBC16 wild-type (WT) and heterozygous (+/W373X) mice of each gender. ‘Other’ represents porphyrins too low in concentration to accurately distinguish. (C) Isomer ratio of faecal coproporphyrin III to I (CIII:CI) and (D) baseline urinary PBG levels measured in female wild-type (WT) and heterozygous (+/W373X) mice. Values are mean±s.d.; *n*=3. **P<0.01, ***P<0.001, n.s., not significant.

The hallmark biochemical feature of HCP is elevated porphyrins, notably coproporphyrinogen III and uroporphyrinogen, in the urine and faeces of affected individuals. Analysis of *Cpox^+/W373X^* female mice, using HPLC, revealed a three- to four-fold increase in porphyrins, particularly coproporphyrinogen III, in the urine (Fig. 3A) and faeces (Fig. 3B) in comparison to wild-type mice. Male heterozygotes, however, did not show elevated porphyrins, and were subsequently excluded from further tests. In the faeces, the ratio of the two coproporphyrinogen isomers (CIII:CI) was significantly elevated in female heterozygotes (Fig. 3C), which is highly specific for the diagnosis of HCP (Allen et al., 2005). Despite elevated levels of porphyrins, urinary porphobilinogen (PBG) was normal in female *Cpox^+/W373X^* mice (Fig. 3D), suggesting a dormant HCP phenotype. Thus, the *Cpox^+/W373X^* mouse strain had some, but not all, of the biochemical features of hereditary coproporphyria.

### Fasting does not induce crisis

**Fig. 4.**
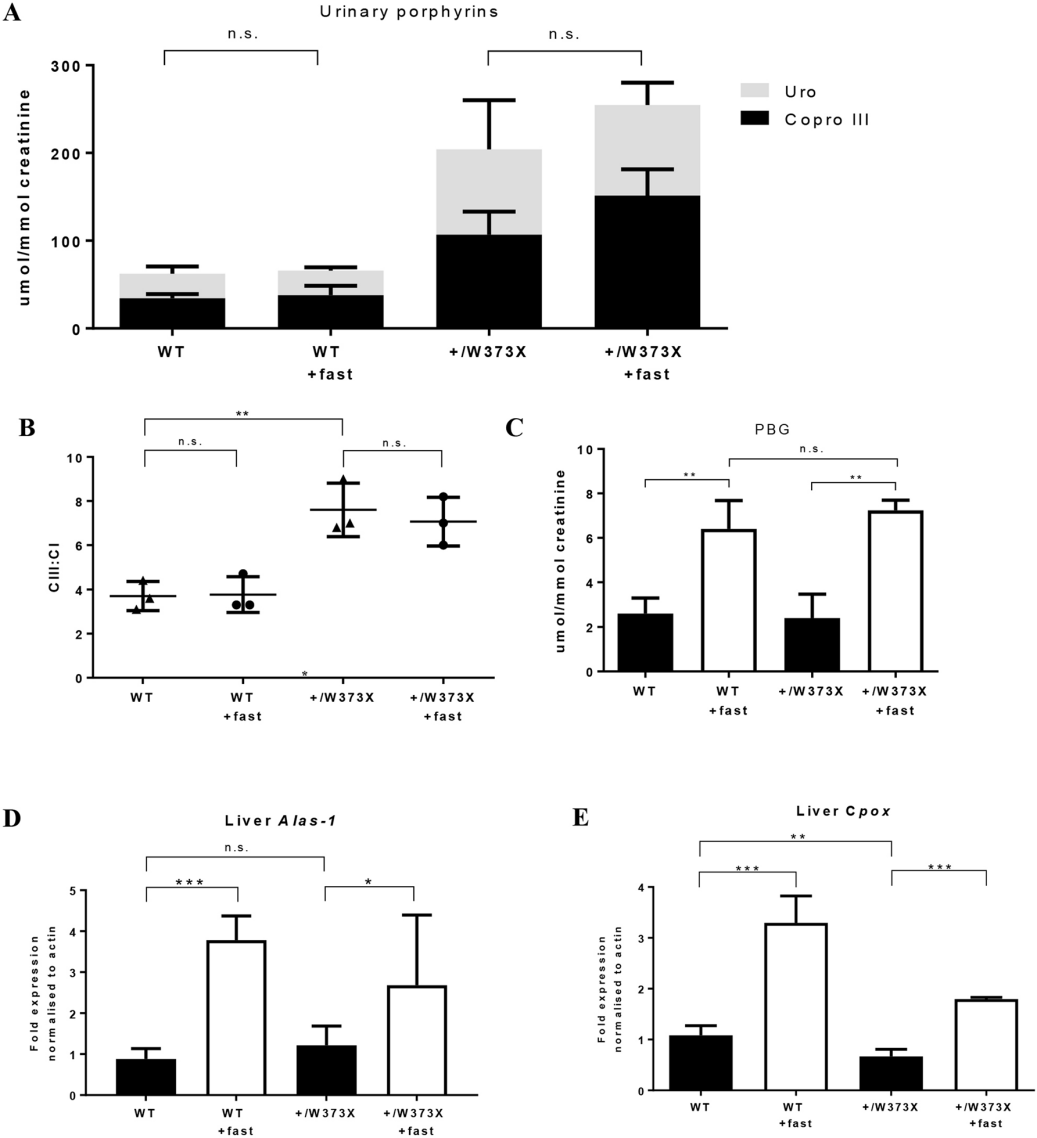
**Effects of fasting on female *Cpox^+/W373X^* mice.** (A) Urinary porphyrins of 4-month-old RBC16 wild-type (WT) and heterozygous (+/W373X) female mice measured before and after (+fast) fasting period. (B) Isomer ratio of faecal coproporphyrin III to I (CIII:CI) measured before and after (+fast) fasting period. (C) Urinary PBG quantification before and after (+fast) fasting period. (D) Quantitative RT-PCR for *Alas-1* and (E) *Cpox* mRNA expression in the liver before and after (+fast) fasting period. Values are mean±s.d.; *n*=3 for A-C and *n*=4 for D,E. *P<0.05, **P<0.01, ***P<0.001, n.s., not significant.

In patients with porphyria, fasting can trigger an acute crisis by activation of hepatic enzymes. In particular, the activity of the enzyme aminolevulinic acid synthase (ALAS-1) is believed to be a crucial driver of clinical crises by supplying haem-synthesising cells with the rate-limiting porphyrin precursor, 5-aminolevulinic acid (ALA) (Handschin et al., 2005). To determine if fasting increases porphyrin production and induces clinical crises in *Cpox^+/W373X^* mice, females were fasted for 15 h on two consecutive days and urine was collected. Despite fasting, mice remained clinically well and urinary porphyrins (uroporphyrinogen and coproporphyrinogen III) did not significantly increase (Fig. 4A). In the faeces, the CIII:CI ratio also remained unchanged (Fig. 4B). There was a three-fold increase in the porphyrin precursor, PBG, in the urine but this was observed equally in wild-type and *Cpox^+/W373X^* mice (Fig. 4C). To understand why fasting did not increase porphyrins, liver mRNA transcription levels of *Alas1* and *Cpox* were analysed via quantitative real-time (Q)PCR. As expected, fasting induced a three- to four-fold increase in *Alas1* expression in both wild-type and *Cpox^+/W373X^* mice (Fig. 4D). *Cpox* mRNA levels also increased proportionally, with fasting levels in *Cpox^+/W373X^* mice remaining approximately half that of wild-type mice (Fig. 4E). Thus, fasting did not induce the clinical or biochemical changes of a porphyric crisis.

### Phenobarbital elevates faecal coproporphyrinogen III in *Cpox^+/W373X^* mice

**Fig. 5.**
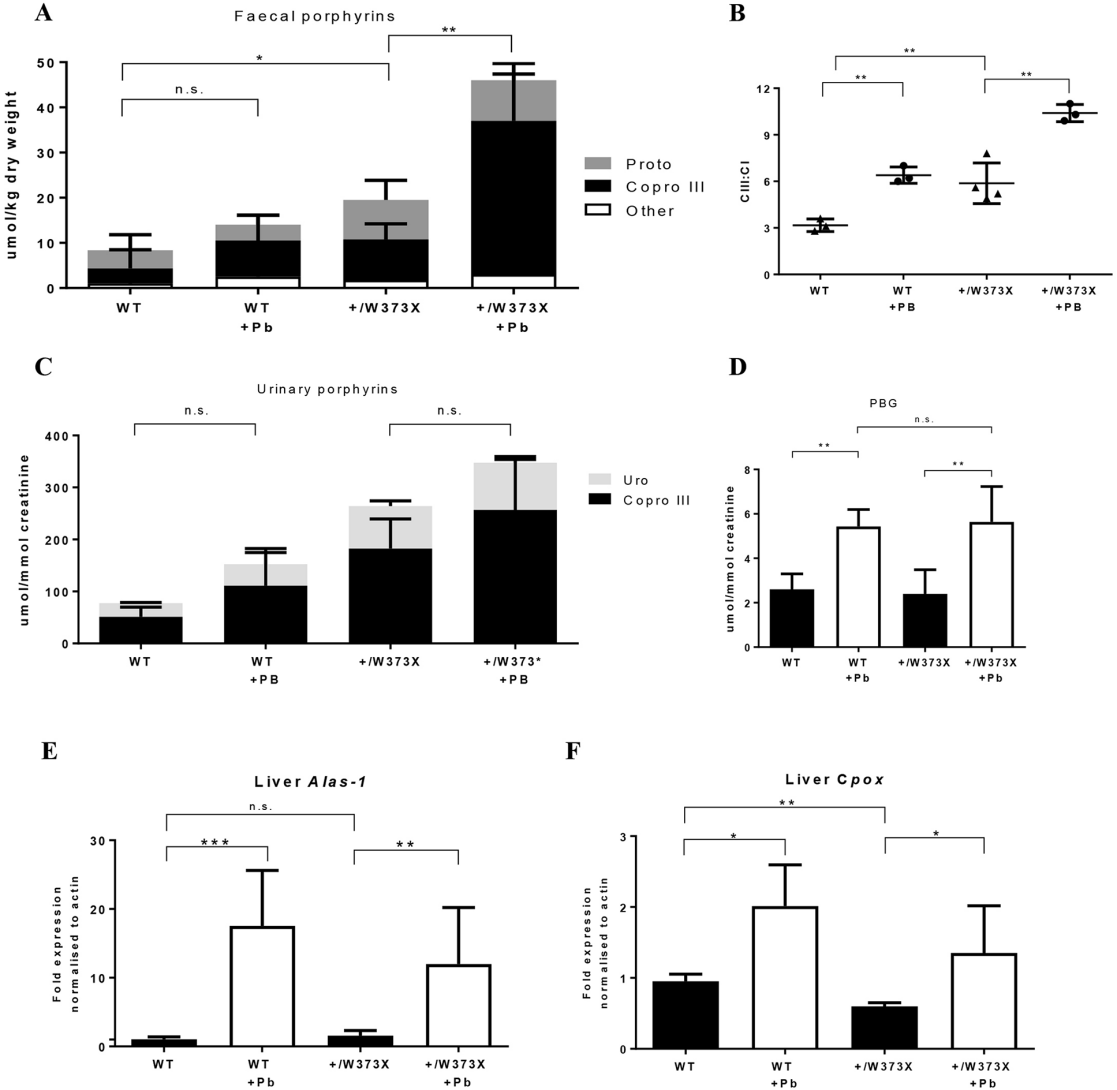
**Effects of phenobarbital treatment on female *Cpox^+/W373X^* mice.** (A) Faecal porphyrins of 4-month-old wild-type (WT) and heterozygous (+/W373X) female mice measured before and after (+Pb) phenobarbital treatment. ‘Other’ represents porphyrins too low in concentration to accurately distinguish. (B) Isomer ratio of faecal coproporphyrin III to I (CIII:CI) measured before and after (+Pb) phenobarbital. (C) Urinary porphyrins of wild-type (WT) and heterozygous (+/W373X) female mice measured before and after (+Pb) phenobarbital treatment. (D) Urinary PBG quantification of wild-type (WT) and heterozygous (+/W373X) female mice measured before and after (+Pb) phenobarbital treatment. (E) Quantitative RT-PCR for *Alas1* and (F) *Cpox* mRNA expression in the liver before and after (+Pb) phenobarbital treatment. Values are mean±s.d.; *n*=3 for A-C and *n*=4 for D,E. *P<0.05, **P<0.01, ***P<0.001, n.s., not significant.

Phenobarbital is a potent porphyrinogenic agent and common crisis stimulant, used in experimental animal models to induce acute porphyric attacks in various porphyria subtypes (Lindberg et al., 1996; Yasuda et al., 2014). Therefore, to induce the maximum porphyric stress, female *Cpox^+/W373X^* mice and age-matched wild-type females were treated daily with phenobarbital for 4 days, followed by analysis of urinary and faecal porphyrins. Faecal coproporphyrinogen III levels in the faeces were significantly elevated in heterozygous *Cpox^+/W373X^* mice following exposure to phenobarbital (Fig. 5A), accompanied by a significant elevation in the CIII:CI ratio (Fig. 5B). However, urinary porphyrins were not significantly increased above baseline levels following phenobarbital treatment (Fig. 5C). Urinary PBG showed an identical three-fold increase in both wild-type and *Cpox^+/W373X^* mice (Fig. 5D). Phenobarbital had a different effect on induction of *Alas1* and *Cpox* mRNA compared with fasting, with a more potent activation of liver mRNA expression of *Alas1* (10- to 20-fold) than *Cpox* (two-fold) (Fig. 5E,F). In summary, the more potent induction of *Alas1* by phenobarbital was able to increase faecal coproporphyrin III in *Cpox^+/W373X^* mice.

## DISCUSSION

The prosthetic haem molecule is a vital component of haemoglobin, myoglobin and cytochromes. Haem is synthesised largely in the bone marrow, but also in hepatocytes and other cells, through a series of linear irreversible enzymatic reactions that occur in the cytosol and mitochondria. Coproporphyrinogen III oxidase (CPOX) is the sixth enzyme in the haem biosynthesis pathway, responsible for the conversion of coproporphyrinogen III into protoporphyrinogen IX. Mutations that aberrantly affect the function or production of this enzyme result in the manifestation of the disease hereditary coproporphyria (HCP), one of the acute hepatic porphyrias (Kauppinen, 2005). Using ENU mutagenesis, we have generated a novel mouse mutant, *Cpox^+/W373X^*, harbouring a base-pair mutation in *Cpox* that results in a premature stop codon in place of a tryptophan at amino acid 373 of exon 5. cDNA analysis and mRNA expression levels suggest this mutation likely results in the degradation of the truncated transcript, therefore reducing the total CPOX protein by 50% in the heterozygote. Anaemia manifests due to insufficient haem availability, while porphyrins, such as uroporphyrinogen and coproporphyrinogen III, are excreted in the urine and faeces in excess because of a blockage in the haem biosynthesis pathway. The *Cpox^+/W373X^* mouse presents with biochemical abnormalities that are specific for the diagnosis of HCP, including an increase in the ratio of coproporphyrin III:I isomers, but does not display an increase in early porphyrin precursors, namely PBG. This suggests that *Cpox^+/W373X^* mice display a latent porphyric phenotype. Interestingly, these biochemical changes were only witnessed in female *Cpox^+/W373X^* mice, which excrete far more porphyrins than age-matched males with an identical mutation. This disparity mimics what is observed clinically in many porphyrias, where female patients often endure acute attacks more frequently and with greater severity than males with the same mutation due to the periodic flux of progesterone levels (Andersson et al., 2003).

Fasting is known to be capable of precipitating symptoms of acute crises in porphyria as a result of the direct activation of ALAS-1 (Handschin et al., 2005). After two consecutive bouts of overnight fasting, *Cpox^+/W373X^* female mice did not show any signs of a clinical crisis, and biochemical analysis of porphyrins and porphyrin precursors showed no significant changes. The mRNA expression levels of *Alas1* and *Cpox* appear central to this observation. Fasting induced an expected increase in *Alas1* expression, which provides more rate-limiting porphyrin precursor (ALA) to haem-synthesising cells. At the same time, there was a proportional increase in *Cpox* mRNA. Working under the hypothesis that the W373X mutation does not generate a functional mutant protein, the increase in wild-type CPOX enzyme, matching increased ALA availability, is likely to have inhibited a subsequent build-up of porphyrins and prevented an acute crisis from developing in *Cpox^+/W373X^* mice. An increase in CPOX enzyme activity may also have contributed to these findings, which remains to be determined. Phenobarbital was used as a more acute porphyrinogenic agent and was able to precipitate some, but not all, biochemical signs of a porphyric crisis. Following 4 days of increasing doses of phenobarbital, the level of faecal coproporphyrinogen III was significantly elevated, along with a further increase in the CIII:CI ratio. In the urine, the change in porphyrins was less notable, and PBG remained normal. On a molecular level, we once again observed how changes in *Alas1* and *Cpox* transcription could have influenced these observations. *Alas1* mRNA expression was dramatically amplified following phenobarbital treatment – 10- to 20-fold greater than pre-treated levels – while *Cpox* mRNA transcription saw only a two-fold increase. This disproportionate production of the ALA precursor, met by insufficient CPOX enzyme, is likely to be linked to the excessive build-up of faecal coproporphyrinogen III witnessed in *Cpox^+/W373X^* female mice. The minimal effect of phenobarbital treatment on urinary porphyrins is still under investigation. The *Cpox^+/W373X^* mouse strain is therefore susceptible to some, but not all, porphyrinogenic triggers, the outcome of which appears to be linked to ALAS-1 activity.

The haematological abnormalities present in the *Cpox^+/W373X^* mouse strain represents an unexpected phenotype that is not typically identified in HCP. Haem deficiency was evident in the *Cpox^+/W373X^* mice. This is known to suppress globin synthesis (Iolascon et al., 2009), resulting in microcytic red cells that lack sufficient haemoglobin, while splenomegaly is induced as a compensatory mechanism of extramedullary erythropoiesis. The additional increase in serum ferritin levels in heterozygotes represents a defect in iron-to-haem loading that requires further investigation. As ENU mutagenesis results in many mutations in experimental animals (Hitotsumachi et al., 1985), it is possible that additional genetic loci are responsible for the haematological phenotype witnessed in the *Cpox^+/W373X^* mouse strain. This could be further investigated by crossing the *Cpox^+/W373X^* mouse onto multiple genetic backgrounds, to determine whether the red cell phenotype, as well as its responsiveness to porphyrinogenic triggers, is genetically transferable. With regards to the embryo lethality observed in homozygous *Cpox^W373X/W373X^* mutants, while red blood cells were visible in the yolk sac, it is likely that they are functionally deficient, although this may be adjacent to the gross developmental irregularities seen in day 9.5 homozygotes. Embryonic growth retardation, including limb and cranial abnormalities, has been observed in murine models with haem iron defects that diminish the free haem pool (Keel et al., 2008). This suggests that haem plays a non-erythropoietic role in embryogenesis, which may contribute to the developmental abnormalities and lethality observed in *Cpox^W373X/W373X^* embryos. It also interesting to note that the *Cpox^+/W373X^* mouse does not present with a hereditary cataract phenotype, in comparison to the established Nakano mouse model, which harbours a missense mutation in *Cpox* with autosomal recessive inheritance and presents with increased accumulation of coproporphyrinogen III in the lenses of homozygous mice (Nakano et al., 1960). While the underlying mechanism defining porphyrins and lens pathology remains unknown in this model, it again suggests that haem plays a variety of roles in physiological development that are yet to be fully determined.

The heterogeneity of HCP in humans and lack of a genotype-phenotype relationship is still a major barrier in predicting patient symptoms and disease severity (Lamoril et al., 2001). This makes the establishment of animal models of HCP difficult, but necessary. Here, we report the first murine model of hereditary coproporphyria, the *Cpox^+/W373X^* mouse strain, with biochemical parallels to the human disease and a haematopoietic phenotype. This mutant model would be beneficial in studies of the molecular basis of porphyrinogenic triggers and haem biosynthesis, and also for the testing of future treatment options to improve the clinical management of the acute hepatic porphyrias.

## MATERIALS AND METHODS

### Mice

A G1 pedigree (RBC16; Cpox^W373X^) displaying microcytosis was identified in an ENU mutagenesis screen as described previously (Brown et al., 2013). Cpox^W373X^ mice were genotyped by PCR amplification of the region spanning the W373X mutation using genomic DNA and the primers: For (5′-TTC TCT GCT GCC CGT TCT AGG T-3′) and Rev (5′-CAG GGG TTG TGC ATA AGA GGT C-3′); followed by sequencing using the Big Dye Terminator reagents to identify the point mutation. All animal experiments were approved by the Animal Ethics Committee of the Alfred Medical and Research Education Precinct and Monash University.

### Gene mapping and next-generation sequencing

Gene mapping, massively parallel sequencing, and bioinformatics of RBC16 was performed by the Australian Genome Research Facility. Mapping was performed by outcrossing affected heterozygotes with wild-type Balb/c mice. Tail genomic DNA of G2 and subsequent generations were subjected to simple sequence length polymorphism (SSLP)-based genome-wide scanning and SNP fine-mapping. The candidate interval was refined to 56.46-60.98 Mb on chromosome 16. A genomic custom capture array was generated by Roche-NimbleGen to preferentially enrich for the genomic sequence within this range. Genomic DNA was extracted from a liver sample harvested from a presumed RBC16 heterozygote, phenotyped based on low MCV and low MCH on full blood count. Massively parallel sequencing was performed using the Illumina HiSeq platform and assembly of reads to the NCBI37/mm9 C57BL/6 reference sequence, which identified a G-to-A point mutation at position c.1118 of *Cpox* (NM_007757.2) resulting in a tryptophan to stop codon substitution at amino acid 373.

### Whole blood analysis

Blood was extracted from mice via submandibular vein bleeds and stored in EDTA tubes. Full blood examination was performed on an automated Hemavet (Drew) blood analyser. Whole blood haem was quantified using the BioAssay QuantiChrom Haem Assay Kit performed in a 96-well plate and read on a Multiskan GO (Thermo Fisher Scientific) plate reader. Serum ferritin was measured by the Mouse Ferritin ELISA Kit (Abcam) performed in a 96-well plate and read on a Multiskan GO (Thermo Fisher Scientific) plate reader.

### Real-time Q-PCR

Total RNA was extracted from liver using TRIzol (Invitrogen) according to the manufacturer's instructions, followed by cDNA amplification of 1 μg total RNA using the Reverse Transcription Kit (Promega). Q-PCR was performed on a LightCycler 480II (Roche Diagnostics) using the GoTaq qPCR Master Mix (Promega). Expression of genes was normalised to β-actin and data are presented as relative expression compared with the wild-type controls. Gene-specific primers were as follows: *Cpox*, For: 5′-CGG AGG ACA TGA AGA CCA AGA T-3′ and Rev: 5′-TGA TGC CAC CTC CTC CTT CT-3′; *Alas1*, For: 5′-TCT TCC GCA AGG CCA GTC T-3′ and Rev: 5′-TGG GCT TGA GCA GCC TCT T-3′.

### Porphyrin and PBG quantification

Mice were housed in individual metabolic cages for the collection of urine and faeces. At least 1 ml urine and 0.5 g faeces was collected from each animal and frozen prior to testing. Urinary porphyrins were measured by a modification of the method of Lim and Peters (1984). Urine (750 μl) was acidified with 50 μl of a solution containing the internal standard deuteroporphyrin. Samples were then vortex mixed, filtered and 60 μl was injected onto a Waters octadecyl reverse-phase column. The samples were run on an Agilent 1200 HPLC system using gradient elution with fluorimetric detection. All porphyrin fraction peaks eluted within 12 min and the total amount was calculated against a six-peak porphyrin calibration standard obtained from Frontier Scientific (Utah, USA). Faecal porphyrins were measured by a modification of the method of Lockwood et al. (1985). A small sample of faeces was homogenised in concentrated HCl and then extracted with ether to remove interfering coloured compounds. On addition of water, carotenoid and chlorophyll derivatives and other coloured compounds move into the ether phase leaving the porphyrins in the acid aqueous phase. The aqueous phase was then scanned on a Varian Cary Spectrophotometer between 350 and 450 nm and the porphyrin concentration of the extract is proportional to the height of the Soret peak which, along with the percentage dry weight of the faeces, was used to calculate the amount of total faecal porphyrin present. PBG was measured as per Blake et al. (1992), where an anion exchange resin was used for the clean-up of urine specimens, followed by the addition of Ehrlich's reagent prior to spectrophotometric absorbance measurement at 553 nm.

### Red cell half-life

Red cell half-life was studied *in vivo* using EZ-Link NHS-Biotin (Thermo Fisher Scientific). Mice were injected intravenously with biotin diluted in a 10% DMSO/90% saline solution at a concentration of 30 mg/kg body weight. Tail bleeds were performed on subsequent days and analysed by flow cytometry. 1-2 μl of blood was stained with FITC-Ter119 and Streptavidin-PE, and the percentage of biotin-positive cells was calculated as a fraction of the total biotin-positive cells on day 1. All antibodies were purchased from BD Sciences and flow cytometry was performed on FACSCalibur (BD Sciences).

### Phenobarbital treatment

Phenobarbital methods were adapted from Johansson et al. (2003). Female mice underwent overnight fasting prior to treatment for 15 h, followed by four daily intraperitoneal injections of phenobarbital (in the form of phenobarbitone) (Aspen Pharma) at increasing concentrations (75, 80, 85, 90 mg/kg body weight). Mice were housed in metabolic cages during treatment for urine and faeces collection, and were sacrificed 4 h after the last dose on day 4 for RNA extraction.

### Statistical analysis

Where applicable, results are expressed as mean±s.d. For statistical analysis, a two-tailed Student's *t*-test was employed, unless stated otherwise; *P<0.05, or as defined in the figure legends.
